# Imaging Synaptic Density in Aging and Alzheimer Disease with [^18^F]SynVesT-1

**DOI:** 10.2967/jnumed.124.269005

**Published:** 2025-04

**Authors:** Joseph Giorgio, David N. Soleimani-Meigooni, Mustafa Janabi, Suzanne L. Baker, Xi Chen, Tyler N. Toueg, Robby Weimer, Bastian Zinnhardt, Ari Green, Gil D. Rabinovici, William J. Jagust

**Affiliations:** 1 Department of Neuroscience, University of California Berkeley, Berkeley, California;; 2 School of Psychological Sciences, College of Engineering, Science, and the Environment, University of Newcastle, Newcastle, New South Wales, Australia;; 3 Memory and Aging Center, Department of Neurology, Weill Institute for Neurosciences, University of California, San Francisco, California;; 4 Lawrence Berkeley National Laboratory, Berkeley, California;; 5 Department of Psychology, Stony Brook University, Stony Brook, New York;; 6 Genentech, Inc; and; 7 Roche Switzerland, Basel, Switzerland

**Keywords:** Alzheimer disease, synaptic density, PET modeling

## Abstract

Synaptic density imaging with PET is a relatively new approach to monitoring synaptic injury in neurodegenerative diseases. However, there are remaining technical and clinical questions, including questions on reference region selection and on how specific phenotypic presentations and symptoms of Alzheimer disease (AD) are reflected in alterations in synaptic density. **Methods:** Using a synaptic vesicle glycoprotein 2A (SV2A) PET ligand radiolabeled with the ^18^F isotope ([^18^F]SynVesT-1), we performed sensitivity analyses to determine the optimal reference tissue modeling approach to derive whole-brain ratio images. Using these whole-brain images from a sample of young adults, older adults, and patients with varied phenotypic presentations of AD, we then contrasted regional SV2A density and in vivo AD biomarkers. **Results:** Reference tissue optimization concluded that a cerebellar gray matter reference region is best for deriving whole-brain ratio images. Using these images, we found a strong inverse association between [^18^F]SynVesT-1 PET uptake and amyloid β and tau PET deposition. Finally, we found that individuals with a lower temporal gray matter volume but higher temporal [^18^F]SynVesT-1 PET uptake show preserved performance on the mini-mental state examination. **Conclusion:** [^18^F]SynVesT-1 PET shows a close association with in vivo AD pathology, and preserved SV2A density may be a possible marker for resilience to neurodegeneration.

## Visual Abstract

**Figure:**
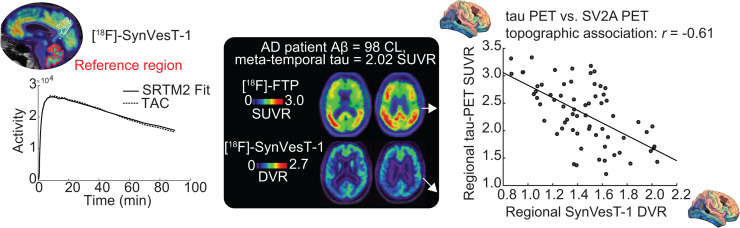

Alzheimer disease (AD) is characterized by the presence of amyloid β (Aβ) and tau deposits, each of which has a profound effect on the number of functional synapses in the brain (1). Recently, PET ligands derived from the structure of levetiracetam, an antiepileptic drug that selectively binds to synaptic vesicle glycoprotein 2A (SV2A), were created to measure in vivo SV2A. The most widely utilized SV2A PET ligand is (4*R*)-1-{[3-(^11^C)methylpyridin-4-yl]methyl}-4-(3,4,5-trifluorophenyl)pyrrolidin-2-one ([^11^C]UCB-J), which exhibits the ideal characteristics of a PET tracer (2–4). However, clinical application of [^11^C]UCB-J is inherently limited by the short half-life of the ^11^C isotope. Subsequent SV2A PET ligands radiolabeled with the ^18^F isotope have been developed, with [^18^F]SynVesT-1 showing similar characteristics to [^11^C]UCB-J (5,6). SV2A PET has been shown to be lower in patients with AD than in controls and is associated with variations in cognition, glucose hypometabolism, Aβ PET, and tau PET (7–17); however, only one of these research groups used [^18^F]SynVesT-1 (17). Further, these prior studies typically investigated amnestic presentations of AD or mild cognitive impairment, and it thus remains to be seen how SV2A PET binding varies across nonamnestic or atypical AD phenotypes.

To relieve the burden on participants, noninvasive reference tissue modeling of PET data is preferred, incorporating limited imaging windows to calculate SUV ratio (SUVR) images. Existing studies have used several different reference regions, including the centrum semiovale and whole cerebellum (Cereb_whole_) for this purpose with this class of compounds (3,17–21). Here, we deployed [^18^F]SynVesT-1 PET in a sample of young adults (Y), cognitively normal older adults (O), and clinically impaired patients with varied AD phenotypic presentations. Using dynamic [^18^F]SynVesT-1 acquisitions, we tested how well a simplified reference tissue model (SRTM) fits captured cortical time–activity curves for various reference regions and examined different imaging time frames for SUVR calculation. We then tested associations between [^18^F]SynVesT-1 PET uptake, Aβ PET, tau PET, and cognitive impairment.

## MATERIALS AND METHODS

### Participants

Twenty-eight participants were selected from 3 populations recruited from 2 independent sites. Seven Y and 14 O were recruited through the Berkeley Aging Cohort Study. Seven patients with clinical presentations of AD were recruited from the University of California San Francisco Memory and Aging Center. These patients had mixed phenotypes: amnestic dementia (*n* = 3), logopenic variant of primary progressive aphasia (*n* = 1), posterior cortical atrophy (*n* = 1), amnestic mild cognitive impairment (*n* = 1), and nonamnestic mild cognitive impairment (*n* = 1). In total, 43% of O and 86% of patients had visually positive or elevated Aβ PET scans (1 amnestic mild cognitive impairment was Aβ-negative) (Table 1). Y participants did not undergo Aβ or tau PET. This study was approved by the relevant Institutional Review Boards of the University of California, Berkeley and San Francisco, and the Lawrence Berkeley National Laboratory (and all subjects gave written informed consent).

**TABLE 1.** Participant Demographics

Demographic	Y	O	Patients
Sample size	7	14	7
Age (y)	26.1 ± 3.8	80.2 ± 4.4	66.4 ± 7.5
Female	4	8	2
Education (y)	19 ± 1.6	17.4 ± 1.3	17.5 ± 2.8
MMSE	29 ± 1.2	28.6 ± 1.5	23.6 ± 9
Aβ-positive	NA	6	6
AD tau–positive	NA	4	5

NA = not applicable.

Qualitative data are number; continuous data are mean ± SD.

### Imaging Acquisition

#### MRI

All participants had a T1-weighted structural MRI (supplemental methods: “Structural MRI”; supplemental materials are available at http://jnm.snmjournals.org) that was segmented into gray matter, white matter, and cerebrospinal fluid components in native space using Statistical Parametric Mapping 12. MR images were additionally parcellated into regions of interest (ROIs) with FreeSurfer version 5.3.0 using the Desikan–Killiany atlas (22).

#### Aβ and Tau PET

All O and patients underwent Aβ and tau PET (supplemental methods: “Aβ and Tau PET”). A global Aβ index was derived using the centiloid scale (23). Tau PET SUVR images were created using an inferior cerebellar gray matter reference region, and from these whole-brain images, average values from the MRI parcellated ROIs were extracted.

#### [^18^F]SynVesT-1 PET

[^18^F]SynVesT-1 was synthesized at the Biomedical Isotope Facility at Lawrence Berkeley National Laboratory, and all participants underwent a dynamic PET acquisition on a Siemens Biograph PET/CT scanner for 90 min across 35 dynamic frames (4 × 15, 8 × 30, 9 × 60, 2 × 180, 10 × 300, and 2 × 600 s). Two O did not complete the full acquisition (33 and 34 frames) (supplemental methods: “[^18^F]SynVesT-1 PET Acquisition”).

### Optimizing Modeling of [^18^F]SynVesT-1 PET

#### Reference Region Selection

We extracted SUVs from each reference region (supplemental methods: “Modeling of [^18^F]SynVesT-1 PET”): cerebellar gray matter (Cereb_GM_), Cereb_whole_, and eroded white matter (WM_eroded_) from the averaged 70- to 90-min frames. We assessed SUV variability with the coefficient of variation (ratio of population SD and mean). Using the SRTM2 (24) and time–activity curves from 34 bilateral cortical Desikan–Killiany ROIs, we derived values for tracer clearance from the reference tissue (k_2_′) for each reference ROI. Using SRTM2, we generated cortical time–activity curves and calculated the average sum squared error (SSE) across ROIs to compare model fit using different reference regions. We determined an optimal reference region by a low average SSE between observed and fit time–activity curves, as well as a low coefficient of variation of SSE, k_2_′, and SUV.

#### Deriving Ratio Images

We next used the group-averaged k_2_′ values to derive whole-brain distribution volume ratio (DVR) images with Logan graphical analysis over 35–90 min of data (25). We calculated the slope of Logan X and Logan Y values over 35–90 and 70–90 min after injection to examine steady state, and we compared slopes for nonlinearities.

To determine optimal SUVR imaging windows, we assessed 3 sets of frames (50–70 min, 60–80 min, and 70–90 min), extracting average uptake bilaterally within cortical ROIs and comparing these with values extracted from the DVR image. We calculated the shared variance between the ROI values, treating each ROI for each subject as an observation. Second, we assessed the within-subject differences in shared variance, treating the within-subject shared variance between ROI uptake as a within-subject repeated observation.

#### Partial-Volume Correction (PVC)

We used a 2-tissue-compartment PVC approach (supplemental methods: “Modeling of [^18^F]SynVesT-1 PET”) (26).

### Associating [^18^F]SynVesT-1 Uptake with Clinical and Imaging Variables

We extracted average tracer uptake from the DVR and SUVR images in 4 summary ROIs: temporal meta-ROI (volume-weighted average of the entorhinal, amygdala, parahippocampal, fusiform, inferior temporal, and middle temporal ROIs) (27), temporal lobe, parietal lobe, and frontal lobe. We a priori selected the temporal meta-ROI as it has been shown to be sensitive to AD-related changes in volume (28) and pathology (tau) (27); our limited sample size precluded more exploratory regional analyses. We contrasted average uptake within these regions for each diagnostic group (Y, O, and patients). Further, we tested the association between Aβ centiloid levels and global cortical [^18^F]SynVesT-1 uptake for O and patients who were Aβ-positive based on a visual read.

Next, we assessed the correlation between [^18^F]SynVesT-1 and tau PET uptake across 34 left and 34 right cortical Desikan–Killiany ROIs (68 total) within each participant using the PVC [^18^F]SynVesT-1 DVR image because 2-tissue-compartment PVC will account for within-subject regional atrophy that will confound the spatial association between [^18^F]SynVesT-1 and tau PET uptake. This method allowed assessment of each tau PET ligand separately.

Finally, we examined whether [^18^F]SynVesT-1 uptake modified the relationship between regional gray matter volume and cognitive performance measured with the mini-mental state examination (MMSE). We use multiple linear regression to model the effects of the temporal meta-ROI [^18^F]SynVesT-1 uptake, temporal meta-ROI gray matter volume, and their interaction on MMSE, including age as a confounding variable. Temporal meta-ROI volume was derived in the same manner as PET ROI derivations and was normalized by total intracranial volume.

Unless otherwise stated, all analyses were repeated using both PVC and non-PVC data.

## RESULTS

### Optimal Reference Region and Acquisition Window for Reference Tissue Images

**FIGURE 1.**
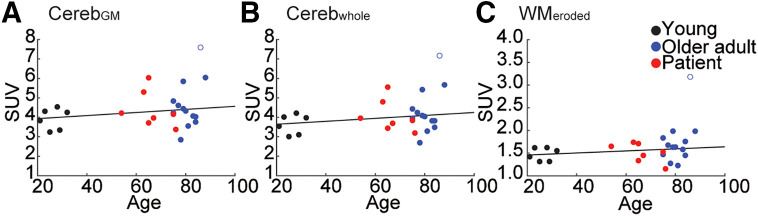
Association between age and SUV for Cereb_GM_ (A), Cereb_whole_ (B), and WM_eroded_ (C). Line shows least-squares best fit.

For the 26 participants who completed 90 min of [^18^F]SynVesT-1 PET acquisition, there was no significant relationship between SUV and age in any reference region (Pearson correlation: Cereb_GM_: *r*_24_ = 0.22, *P* = 0.3; Cereb_whole_: *r*_24_ = 0.23, *P* = 0.28; WM_eroded_: *r*_24_ = 0.24, *P* = 0.26, 1 outlier was removed) (Fig. 1), nor were there statistical differences in SUVs in any reference region when comparing the Y, patient, and O groups (1-way ANOVA: Cereb_GM_: *F*_2,23_ = 0.95, *P* = 0.4; Cereb_whole_: *F*_2,23_ = 0.99, *P* = 0.39; WM_eroded_: *F*_2,23_ = 1.47, *P* = 0.25) (Supplemental Fig. 1). All reference regions had similar coefficients of variation (0.237–0.238), although the uptake in the WM_eroded_ was lower than in the 2 cerebellar reference regions for all but 1 participant (Cereb_GM_ [mean ± SD]: 4.4 ± 1.04; Cereb_whole_: 4.1 ± 0.97; WM_eroded_: 1.62 ± 0.38).

**FIGURE 2.**
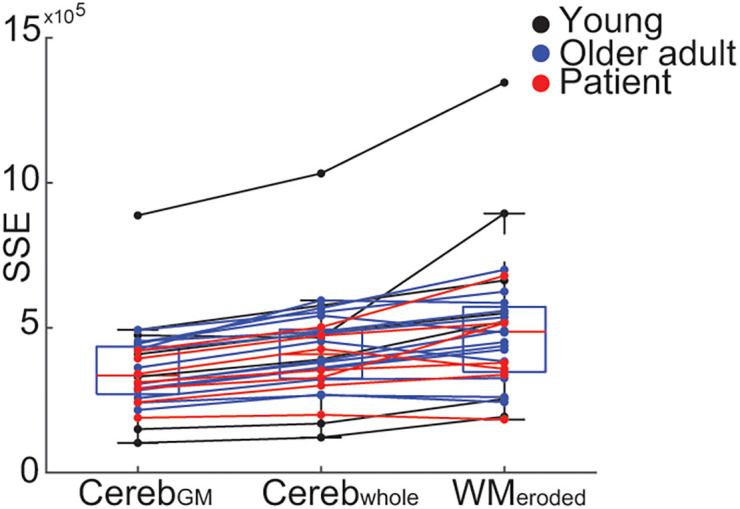
SRTM2 goodness of fit for different reference regions. Within-subject SSE of SRTM2 predicted time–activity curves and observed time–activity curves using Cereb_GM_, Cereb_whole_, or WM_eroded_ reference region.

All k_2_′ values were reliably estimated for each reference region, with low coefficients of variation among the sample (Supplemental Table 1). Furthermore, cortical time–activity curves were fit with similar accuracy to observed time–activity curves among each group, with no significant differences in SSE between Y, patient, or O groups (1-way ANOVA: Cereb_GM_: *F*_2,25_ = 0.7, *P* = 0.51; Cereb_whole_: *F*_2,25_ = 0.52, *P* = 0.6; WM_eroded_: *F*_2,25_ = 1.7, *P* = 0.20). Similarly, we did not observe an effect of age on time–activity curve fit (Pearson correlation: Cereb_GM_: *r*_24_ = 0.22, *P* = 0.3; Cereb_whole_: *r*_24_ = 0.23, *P* = 0.28; WM_eroded_: *r*_24_ = 0.24, *P* = 0.26) (Supplemental Fig. 2). However, using the Cereb_GM_ reference region, the fit of the SRTM2-estimated time–activity curve with the observed time–activity curve showed the least error (1-way repeated-measures ANOVA: *F*_2,54_ = 32.4, *P* < 0.0001; Cereb_GM_ vs. Cereb_whole_ change in SSE: −66,304, *P* < 0.0001, Cereb_GM_ vs. WM_eroded_ change in SSE: −139,630, *P* < 0.0001, Tukey honestly significant difference–corrected) (Fig. 2). Taken together, these results indicate that a Cereb_GM_ reference region is preferred when deriving DVR images; we therefore used Cereb_GM_ as the reference region throughout. In the 26 participants with full emission data, we observed highly similar slopes when fitting the data in the 35- to 90-min or the 70- to 90-min windows (Supplemental Fig. 3), although the slope using the 70- to 90-min window was slightly lower (slope, 35–90 min to 70–90 min, 0.01; *t*_25_ = 2.49, *P* = 0.02). As a difference of 0.01 is only 2.5% of the dynamic range within our sample, we selected the 35- to 90-min window for the derivation of DVRs.

**FIGURE 3.**
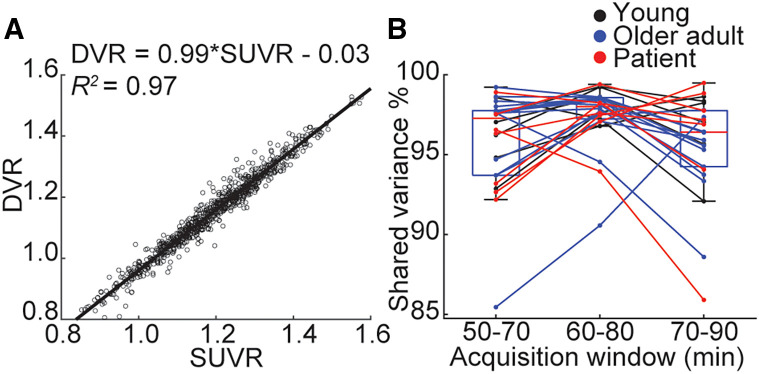
Optimal window for SUVR derivation. (A) Relationship between SUVR and DVR in each cortical ROI for each subject using 60- to 80-min acquisition. Text shows equation of least-squares fit of SUVR to DVR as well as shared variance (*R*^2^) between measures. (B) Within-subject shared variance between SUVR and DVR within cortical ROIs for different windows used for SUVR derivation.

We observed high shared variance (*R*^2^) across all subjects and ROIs between DVR and the different SUVR windows, with the highest occurring in the 60- to 80-min acquisition window (50- to 70-min *R*^2^ = 0.949; 60- to 80-min *R*^2^=0.965; 70- to 90-min *R*^2^=0.959). Calculating the slope of the least-squares fit between DVR and SUVR, we observed a slope closest to 1 (slope, 0.991) in the 60- to 80-min window (Fig. 3). Comparing the percentage of shared variance within subjects showed significant differences between the windows, with the closest association between DVRs and SUVRs observed using a 60- to 80-min acquisition window (1-way repeated-measures ANOVA: *F*_2,50_ = 3.84, *P* = 0.028; 60- to 80-min vs. 50- to 70-min change in *R*^2^ = 1.6%, *P* = 0.006; 60- to 80-min vs. 70- to 90-min change in *R*^2^ = 1.9%, *P* = 0.011; Tukey honestly significant difference–corrected) (Fig. 3). Taken together, these results indicate that an SUVR image derived using emission data 60–80 min after injection is highly representative of data extracted from the full dynamic acquisition. A 60- to 80-min SUVR was used in all subsequent SUVR analyses.

### Differentiating Diagnostic Groups Using [^18^F]SynVesT-1 DVR and SUVR

**FIGURE 4.**
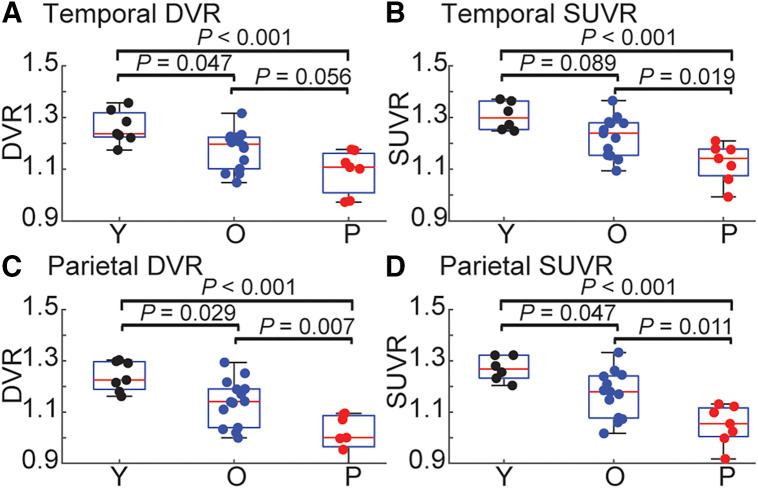
Group differences in DVR (A and C) and SUVR (B and D) in temporal lobes (A and B) and parietal lobes (C and D). Two O did not have PET acquisition within 60- to 80-min window, and SUVR could not be derived. *P* values for groupwise contrasts within each ROI are shown above plots.

For both DVR and SUVR data, the highest uptake was in Y, with intermediate uptake in O and the lowest uptake in patients (Supplemental Fig. 4) (1-way ANOVA: temporal lobe DVR: *F*_2,25_ = 9.21, *P* = 0.004; SUVR: *F*_2,23_ = 10.14, *P* = 0.0007; parietal lobe DVR: *F*_2,25_ = 13.91, *P* < 0.001; SUVR: *F*_2,23_ = 12.31, *P* = 0.0002; post hoc contrasts shown in Fig. 4; temporal meta-ROI DVR: *F*_2,25_ = 6.8, *P* = 0.004; SUVR: *F*_2,23_ = 8.57, *P* = 0.0017; frontal lobe DVR: *F*_2,25_ = 11.16, *P* = 0.0003; SUVR: *F*_2,23_ = 10.52, *P* = 0.0006; post hoc contrasts shown in Supplemental Fig. 5). With PVC, we observed an attenuated difference between Y and O, but patients continued to have lower uptake than O (2-sample *t* test: temporal meta-ROI: *t*_19_ = −2, *P* = 0.06; temporal lobe: *t*_19_ = −2.2, *P* = 0.04; parietal lobe: *t*_19_ = −2.34, *P* = 0.03; frontal lobe: *t*_19_ = −1.896, *P* = 0.073) (Supplemental Fig. 6). Taken together, this suggests there are differences in [^18^F]SynVesT-1 uptake across the cortex between cognitively normal O and patients and that this difference is unlikely to be driven solely by differences in gray matter volume between the groups.

### [^18^F]SynVesT-1 Uptake Mirrors Phenotypic Tau Uptake, Tracks Aβ Levels, and Interacts with Gray Matter Volume When Estimating Cognition

**FIGURE 5.**
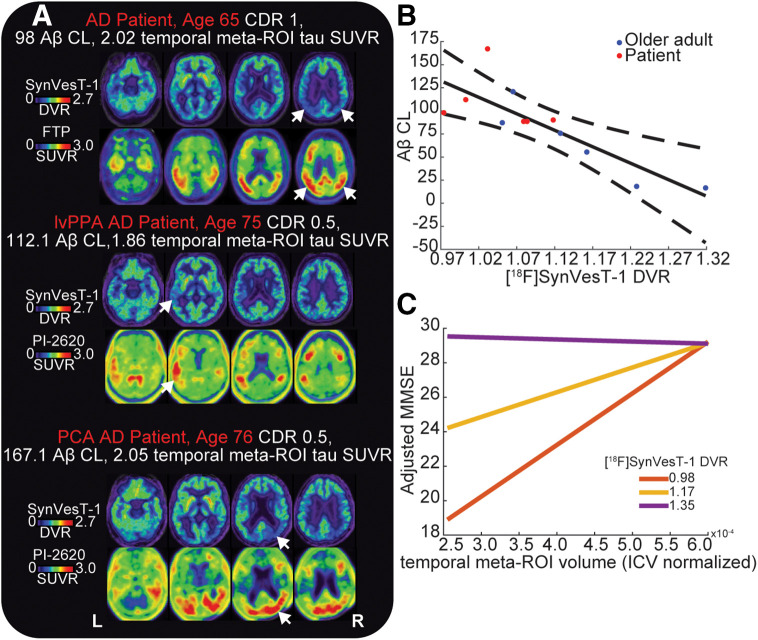
Relationship between [^18^F]SynVesT-1 uptake and AD pathology. (A) [^18^F]SynVesT-1 and tau PET binding patterns for 3 patients with typical amnestic AD (top), logopenic variant of primary progressive aphasia due to AD (middle), or posterior cortical atrophy due to AD (bottom). Arrows show regions of high tau PET uptake (lower panel) and corresponding regions of low [^18^F]SynVesT-1 uptake (upper panel). (B) Association between Aβ centiloid and cortical [^18^F]SynVesT-1 uptake. Solid line is least-squares fit between Aβ centiloid and [^18^F]SynVesT-1; dashed lines are 95% CIs of this fit. (C) Interaction between average temporal meta-ROI volume and [^18^F]SynVesT-1 uptake in predicting MMSE. Individual lines indicate predicted MMSE at different level of temporal meta-ROI volume for 3 different levels of temporal meta-ROI [^18^F]SynVesT-1 uptake: minimum [^18^F]SynVesT-1 DVR in sample (red), average [^18^F]SynVesT-1 DVR in sample (yellow), and maximum [^18^F]SynVesT-1 DVR in sample (purple). MMSEs on *y*-axes are adjusted for age.

We correlated the uptake of [^18^F]SynVesT-1 with the tau PET ligand within each participant, generating a correlation coefficient for each participant. Across the combined O and patient groups, the correlation values were significantly less than 0 (*t*_20_ = −3.5, *P* = 0.002), indicating a negative association between regional binding of the tau tracer and [^18^F]SynVesT-1. Further, we observed that patients had a significantly greater negative association between tau PET and [^18^F]SynVesT-1 uptake than did O (*t*_19_ = −4.76, *P* < 0.001) (Supplemental Fig. 7). Visual inspection of the spatial distributions of [^18^F]SynVesT-1 uptake and tau PET confirmed this mirroring of signal, whereby areas of high tau PET uptake showed low [^18^F]SynVesT-1 uptake. In patients with amnestic dementia due to AD, areas of high bilateral temporoparietal tau PET tracer uptake had lower levels of [^18^F]SynVesT-1 uptake. The patient with a logopenic variant of primary progressive aphasia due to AD had high tau PET uptake in the left temporal cortex, posterior perisylvian, and parietal regions that corresponded to low uptake of [^18^F]SynVesT-1. Finally, we observed a similar mirroring in the patient with posterior cortical atrophy due to AD, who had parietooccipital tau binding on the right greater than that on the left, which corresponded to low [^18^F]SynVesT-1 uptake (Fig. 5).

Next, we tested whether the cortical uptake of [^18^F]SynVesT-1 correlates with Aβ centiloid level in the 12 Aβ participants with a visually positive scan (6 patients, 6 O). We observed a strong negative correlation between centiloid levels of Aβ and average cortical [^18^F]SynVesT-1 uptake without and with PVC (Pearson correlation: without PVC: *r*_10_ = −0.83, *P* < 0.001; with PVC: *r*_10_ = −0.68, *P* = 0.016) (Fig. 5). A multiple regression including the interaction between clinical diagnosis and [^18^F]SynVesT-1 uptake showed no significant interaction or main effects with diagnosis on Aβ centiloid when including [^18^F]SynVesT-1 uptake (adjusted *R*^2^ = 0.61, *P* = 0.014; [^18^F]SynVesT-1: *t* = −3.19, *P* = 0.013; diagnosis: *t* = −0.66, *P* = 0.53; [^18^F]SynVesT-1 × diagnosis: *t* = 0.69, *P* = 0.51) (Supplemental Fig. 8). The significance of regression coefficients was the same using PVC [^18^F]SynVesT-1 uptake.

Using a multiple regression including age as a confounding variable, we modeled the effects of temporal meta-ROI [^18^F]SynVesT-1 uptake, temporal meta-ROI gray matter volume (normalized by total intracranial volume), and their interaction on MMSE. One patient with a low MMSE score was omitted from this analysis (MMSE, 4). This model explained substantial variance in MMSE (adjusted *R*^2^ = 0.6, *P* < 0.001), with significant main and interaction effects (temporal meta-ROI volume: *t* = 2.60, *P* = 0.016; temporal meta-ROI [^18^F]SynVesT-1 uptake: *t* = 2.55, *P* = 0.018; temporal meta-ROI [^18^F]SynVesT-1 uptake × temporal meta-ROI volume: *t* = −2.27, *P* = 0.034) (Fig. 5). Individuals with a low gray matter volume but high [^18^F]SynVesT-1 uptake had relatively preserved cognition (Fig. 5). This result was the same when modeling the interaction using PVC temporal meta-ROI [^18^F]SynVesT-1 uptake (adjusted *R*^2^ = 0.58, *P* < 0.001), with significant main and interaction effects (temporal meta-ROI volume: *t* = 2.41, *P* = 0.025; temporal meta-ROI [^18^F]SynVesT-1 uptake: *t* = 2.33, *P* = 0.03; temporal meta-ROI [^18^F]SynVesT-1 uptake × temporal meta-ROI volume: *t* = −2.06, *P* = 0.05) (Supplemental Fig. 9). Model interpretation and results were the same when Y was excluded, although statistical power was greatly reduced (Supplemental Fig. 9). This further supports the additive information of [^18^F]SynVesT-1 PET relative to MRI atrophy markers and suggests that increased density of SV2A may offset the effects of lower cortical volume on cognition.

## DISCUSSION

In this study of [^18^F]SynVesT-1 PET in a heterogeneous sample, we used reference tissue modeling to show that the Cereb_GM_ provided the optimal characteristics for a reference region. To date, most SV2A PET studies using reference tissue modeling have used the centrum semiovale as a reference region (20). Recently, however, a cerebellar reference region has been used, particularly when investigating synaptic changes in AD (9,12,17,29). Here, we show that the Cereb_GM_ performs better than an WM_eroded_ reference region encompassing the centrum semiovale. Initial reference region selection for SV2A PET using [^11^C]UCB-J determined the centrum semiovale as a preferred reference region (3). Subsequent tracer blocking studies using the antiepileptic drug padsevonil supported this region, showing displaceable binding in cortical, subcortical, and cerebellar regions but no significant difference in centrum semiovale uptake (18). However, additional blocking studies using a different antiepileptic targeting SV2A, levetiracetam, reported a 12%–22% reduction in [^11^C]UCB-J uptake within the centrum semiovale (3,30); this finding was subsequently replicated using [^18^F]SynVesT-1 PET (5). These blocking studies suggest that neither the cerebellum nor the centrum semiovale is a perfect reference region for SV2A PET; however, the Cereb_GM_ may be the preferred reference region based on the consistency of binding across diagnostic groups and the reliability of predictions of time–activity curves using SRTM2.

We observed that [^18^F]SynVesT-1 PET uptake was reduced across the cortex in AD and correlated negatively with Aβ burden in cognitively normal and impaired individuals who were Aβ-positive. Furthermore, the spatial extent of tau pathology characteristic of AD phenotypes is mirrored by reduced [^18^F]SynVesT-1 PET uptake. This extends previous work in typical AD showing that decreases in SV2A on PET are associated with both Aβ (9,10) and tau PET increases (9,11,15,17) and that longitudinal reductions in SV2A PET follow a Braak staging scheme similar to that for tau PET (13). Further, after accounting for gray matter differences in [^18^F]SynVesT-1 PET uptake using PVC, we did not observe significant differences in cortical synaptic density between Y and O, but significant differences between O and patients persisted. The lack of association between SV2A PET in cortical regions and aging replicates previous imaging studies (31–33).

When estimating cognition, we observed that [^18^F]SynVesT-1 PET uptake in the temporal meta-ROI interacted with brain volume, whereby higher synaptic density attenuated the effect of low brain volume on MMSE, implicating synaptic density as a resilience factor. Previous work has shown significant differences in SV2A PET between AD patients and controls (7,9,12,13,15–17,29), as well as associations between temporal SV2A PET uptake and cognition (7,29). Here we extend these findings by detecting this interaction.

There are several limitations to this work. First, the sample size was small within each group, and patients were younger than older controls. However, we observed lower [^18^F]SynVesT-1 PET uptake in patients than in either Y or O, giving us confidence that we could reliably capture disease-related reductions in presynaptic density. Further, it is possible that we observed attenuated differences between patients and O as this sample included a subset with AD pathology. Second, we did not investigate the effect of perfusion variability on the association between DVR and SUVR derived in different windows. Previous work using [^11^C]UCB-J indicated that perfusion differences can confound early-window SUVR derivation, inflating group differences. Future work will be required to understand how perfusion differences relate to group differences in [^18^F]SynVesT-1 ratios (34). Finally, because the Cereb_GM_ shows displaceable binding, terms are no longer cancelled out in the derivation of binding potential using SRTM2, and as such, the DVR no longer represents binding potential plus 1 (35). Therefore, care needs to be taken in the interpretation of values derived from these dynamic analyses. Additional studies using arterial sampling and 1-tissue-compartment modeling may be required to provide a gold standard validation of the Cereb_GM_ as the optimal reference region for [^18^F]SynVesT-1 PET in AD.

## CONCLUSION

Here, we present a modeling study of [^18^F]SynVesT-1 PET paired with an investigation of SV2A variations across aging and AD clinical phenotypes. To facilitate a broader use of [^18^F]SynVesT-1 PET in clinical research, we suggest a Cereb_GM_ reference region and a 60- to 80-min-postinjection PET acquisition window to derive SUVR images. Further, we observed a close negative association between the pattern of [^18^F]SynVesT-1 and tau PET in patients with varied phenotypic presentations of AD, as well as a negative association between global SV2A density and Aβ burden. Finally, we observed that participants with neurodegeneration but preserved SV2A density showed higher cognitive performance, suggesting SV2A density as a possible indicator of resilience.

## DISCLOSURE

This research was funded by an award from the Alliance for Therapies in Neuroscience (ATN, Roche-Genentech, University of California Berkeley and the University of California San Francisco). Joseph Giorgio is supported by the Alzheimer Association (23AARF-1026883). William Jagust serves as a consultant to Biogen, Genentech, CuraSen, Bioclinica, and Novartis. No other potential conflict of interest relevant to this article was reported.

## Notes

KEY POINTS **QUESTION:** Which approaches are optimal to derive reference tissue images of [^18^F]SynVesT-1 PET and assess differences in synaptic density in varied AD phenotypes? **PERTINENT FINDINGS:** We found the Cereb_GM_ reference region to be best at modeling ground truth [^18^F]SynVesT-1 PET time–activity curves. Whole-brain [^18^F]SynVesT-1 PET images showed that regions with high tau PET uptake had lower [^18^F]SynVesT-1 PET uptake. Finally, we found a significant interaction between gray matter volume and [^18^F]SynVesT-1 PET uptake, whereby individuals with a low gray matter volume but high synaptic density showed better cognitive performance. **IMPLICATIONS FOR PATIENT CARE:** Our study shows that [^18^F]SynVesT-1 PET has clinical significance through improving our understanding of synaptic density variation in AD.
